# Partitioning of excess mortality in population-based cancer patient survival studies using flexible parametric survival models

**DOI:** 10.1186/1471-2288-12-86

**Published:** 2012-06-24

**Authors:** Sandra Eloranta, Paul C Lambert, Therese ML Andersson, Kamila Czene, Per Hall, Magnus Björkholm, Paul W Dickman

**Affiliations:** 1 Department of Medical Epidemiology and Biostatistics, Karolinska Institutet, , Box 281, Sweden; 2 Center for Biostatistics and Epidemiology, Department of Health Sciences, University of Leicester, UK; 3 Department of Medicine, Division of Hematology, Karolinska University Hospital Solna, Sweden

**Keywords:** Survival analysis, Cancer, Relative survival, Regression models, Competing risks

## Abstract

**Background:**

Relative survival is commonly used for studying survival of cancer patients as it captures both the direct and indirect contribution of a cancer diagnosis on mortality by comparing the observed survival of the patients to the expected survival in a comparable cancer-free population. However, existing methods do not allow estimation of the impact of isolated conditions (e.g., excess cardiovascular mortality) on the total excess mortality. For this purpose we extend flexible parametric survival models for relative survival, which use restricted cubic splines for the baseline cumulative excess hazard and for any time-dependent effects.

**Methods:**

In the extended model we partition the excess mortality associated with a diagnosis of cancer through estimating a separate baseline excess hazard function for the outcomes under investigation. This is done by incorporating mutually exclusive background mortality rates, stratified by the underlying causes of death reported in the Swedish population, and by introducing cause of death as a time-dependent effect in the extended model. This approach thereby enables modeling of temporal trends in e.g., excess cardiovascular mortality and remaining cancer excess mortality simultaneously. Furthermore, we illustrate how the results from the proposed model can be used to derive crude probabilities of death due to the component parts, i.e., probabilities estimated in the presence of competing causes of death.

**Results:**

The method is illustrated with examples where the total excess mortality experienced by patients diagnosed with breast cancer is partitioned into excess cardiovascular mortality and remaining cancer excess mortality.

**Conclusions:**

The proposed method can be used to simultaneously study disease patterns and temporal trends for various causes of cancer-consequent deaths. Such information should be of interest for patients and clinicians as one way of improving prognosis after cancer is through adapting treatment strategies and follow-up of patients towards reducing the excess mortality caused by side effects of the treatment.

## Background

Observational studies of cancer patient survival often use data recorded by population-based cancer registries and are typically analyzed using relative survival. Relative survival is defined as the observed (all-cause) survival, *S*(*t*), among the cancer patients divided by the expected survival, ^*S*∗^(*t*), in a comparable group (with respect to age, sex, calendar year and possibly other covariates) in the general population. On the hazard scale, relative survival provides a measure of excess mortality that can be assumed to be entirely, directly or indirectly, attributable to the disease [1]. One reason for why modelling excess mortality has become the preferred method for population-based cancer patient survival analysis is that it not only captures deaths that are directly due to the cancer in question but also deaths that can be thought of as indirect or cancer-consequent, without relying on the classification of cause of death. There are, however, research areas of clinical interest that involve estimating the effect of one particular component of the excess mortality and existing methodology does not provide an immediate answer to how such an analysis might be carried out. For example, late adverse health effects in cancer patients is a growing problem given the longer survival seen for most cancers. Cause-specific survival in breast cancer patients is far better today than 20 years ago probably due to intensified mammography screening and more prevalent use of adjuvant therapy such as anti-hormones and chemotherapy. Several studies have, however, identified an increasing risk of cardiovascular disorders, mainly myocardial infarction, possibly associated with radio- and chemotherapy such as anthracyclines, in breast cancer survivors. If the primary interest lies in studying temporal trends in treatment-related mortality following a diagnosis of breast cancer, how can we best identify the deaths that occur as a consequence of the treatment? It is well-known that radiotherapy and chemotherapy following a breast cancer diagnosis cause cardiac dysfunction and increase cardiovascular mortality more than 15 years after diagnosis and hence indirectly contribute to excess breast cancer mortality [2-4]. These deaths are particularly difficult, if at all possible, to identify solely based on the information stated on the death certificate. The reason is that a correct classification of death due to treatment-induced cardiovascular disease (CVD) would require knowledge about which cardiovascular deaths would not have occurred in the absence of a cancer diagnosis. Previous work in this area has involved comparing CVD specific mortality ratios by laterality in women treated with radiotherapy compared to women who did not receive radiotherapy treatment [5] or via modelling of standardized mortality ratios [6]. The first approach is often not appropriate for cancer register data where treatment is, if recorded, not randomized. Moreover, both approaches analyse the excess CVD mortality as an isolated condition, ignoring the fact that the excess CVD mortality is only one component of the excess mortality, and thus the possibility that certain covariate effects can be assumed to be equal for the different component parts. In situations where one of the events is rare such assumptions may become necessary to avoid overfitting the model [7]. Pintilie and others [8] have recently suggested a case-cohort approach to estimating treatment-related mortality in patients diagnosed with Hodgkin lymphoma while simultaneously accounting for competing causes of death by borrowing ideas from Fine and Gray [9]. We suggest an alternative approach, building on work of Royston and Parmar [10] on flexible parametric survival models and later adapted for relative survival by Nelson et al. [11]. The latter models are fitted on the log cumulative excess hazard scale using restricted cubic splines [12] for the baseline excess hazard and for any time-dependent effects. By borrowing ideas from classical competing risks theory and incorporating background mortality rates, stratified by the reported underlying causes of death, reported in the Swedish population we propose a model that simultaneously models the number of CVD-deaths and remaining deaths (i.e., deaths other than CVD deaths) among the cancer patients that occur in excess to what is expected in a cancer-free population.

Crude probabilities of death due to cancer and other causes can be derived using the theory of competing risks and have previously been shown to be particularly useful under circumstances where it is of interest to communicate cancer prognosis while accounting for the fact that cancer patients are at risk of experiencing mortality due to a wide range of other causes than their cancer. Cronin et al. showed how the crude probability of death due to cancer and other causes can be calculated from life-tables [13]. The theory has subsequently been further developed by Lambert et al. to show how the crude probabilities of death can be calculated after fitting a flexible parametric relative survival model to individual patient data [14]. In this paper we show how the excess hazard functions, related to the different outcomes under investigation, can be used further to partition the crude probabilities of death into component parts (i.e., death due to excess CVD or other cancer-related causes).

The proposed methodology is illustrated using women diagnosed with breast cancer in Sweden between 1973-1992 and followed up for a maximum of 15 years. The paper is outlined as follows: The Methods section describes relative survival, flexible parametric models for relative survival and provides a framework for how flexible parametric models are adopted for modelling competing risks. The Results and discussion section describes the breast cancer application, implements the method on this data set, and discusses the assumptions of the models. Potential areas for future development and refinement are discussed in the Conclusions section of the paper.

## Methods

### Excess mortality and relative survival

The total (all-cause) hazard, *h*(*t*), experienced by the patients is the sum of two components, the expected hazard rate in the background population, ^*h*∗^(*t*), and the excess hazard rate, *λ*(*t*), associated with a diagnosis of the cancer, i.e.,

(1) $$h(t)=h^{*}(t)+\lambda (t).$$

The survival analogue of the excess hazard rate is the relative survival and on the survival scale the total (all-cause) survival can be written as

(2) $$S(t)=S^{*}(t)R(t)$$

where ^*S*∗^(*t*) is the expected survival and *R*(*t*) is the relative survival at time *t*. Both ^*S*∗^(*t*) and ^*h*∗^(*t*) are assumed known and are usually obtained from routine data sources (e.g., national or regional life tables).

### Flexible parametric models

Flexible parametric models for survival analysis were developed by Royston and Parmar [10] and extended to relative survival by Nelson et al. [11]. These models are fitted in continuous time on the log cumulative baseline excess hazard scale using restricted cubic splines to estimate the baseline log cumulative excess hazard. In addition, as flexible parametric models are fitted on individual level data, continuous covariates can easily be included. The total (all-cause) cumulative hazard, *H*(*t*), is retrieved by integrating equation 1 to give

(3) $$H(t)=H^{*}(t)+\Theta (t).$$

In the above expression ^*H*∗^(*t*) is the expected cumulative hazard in a comparable group from the background population and assumed known whereas *Θ*(*t*) is the cumulative excess hazard assumed to be attributable to the cancer. By assuming that the cumulative excess hazard is a multiplicative function of the covariates, **x**, and that the effects are proportional with respect to the underlying time scale gives

(4) $$\text{ln}(\Theta (t;x))=s(\text{ln}(t);\gamma_{0},k_{0})+x^{T}\beta .$$

Here the log cumulative baseline excess hazard is represented by restricted cubic splines for ln(t), $s(\text{ln}(t);\gamma_{0},k_{0})$, characterised by the vector of knot positions, **k**_0_, and the vector of parameters associated with the spline variables, *γ*_**0**_ and where the effects of covariates, **x**, are given by ***β***. The derivation of the spline function has been described in detail elsewhere [10].

### Time-dependent effects

Non-proportional excess hazards models, i.e., models with time-dependent covariate effects, are often biologically plausible when studying cancer patient survival and such models can be fitted by including interaction terms for the covariate effects that are assumed to depend on time and the spline function representing the time scale. In flexible parametric models the time-dependent effects generally require fewer knots than the baseline effects [11] so for each time-dependent effect, *x*_*i*_, a new configuration of the knots may be chosen. This gives the model

(5) $$\text{ln}(\Theta (t;x))=s(\text{ln}(t);\gamma_{0},k_{0})+x^{T}\beta +\overset{D}{\underset{i=1}{\sum }}s(\text{ln}(t);\gamma_{i},k_{i})x_{i}$$

where *D* is the number of time-dependent covariate effects and $s(\text{ln}(t);\gamma_{i},k_{i})$ is the spline function for the *i*^*th*^ time-dependent effect. Note that for each of the *D* time-dependent effects represented by *x*_*i*_ in the model above are typically a subset of **x**.

Flexible parametric survival models have advantages over Poisson regression models for excess mortality, which fit piecewise constant effects for the baseline excess hazard rate, as they obviate the need for splitting the time scale into a number of intervals. In contrast to equation 5, a piecewise approach, with a reasonable number of split points, typically implies estimating a large number of parameters for the time-dependent effects. A number of alternative approaches to model *λ*(*t*) have however also been proposed [15-18]. While the most common solution for handling time-dependent covariate effects is via inclusion of interaction terms between the covariates that depend on time and the time-scale, alternative approaches for assessing time-dependence and goodness-of-fit have also been proposed [18,19].

In the current application of flexible parametric models the outcome is mortality. For this reason we will use the term *excess mortality*, in place of *excess hazard*. The latter is, in most applications of survival analysis, regarded as the generic term for any time-to-event outcome and the reader may think of the terms as being exchangeable.

### Flexible parametric models for component-specific excess mortality

In order to partition the excess mortality into component parts, the ability to identify the excess mortality related to the specific cause of interest in the data is central. Treatment-related side effects leading to death contribute to excess mortality in cancer patients and, in particular, excess CVD mortality has been reported in women with breast cancer [3,5]. These type of events are difficult to identify solely based on death certificate information but an estimate of the excess mortality caused by the treatment can be retrieved indirectly by comparing the CVD event rate in the group of patients to that in a comparable group, assumed to be free from the cancer in question, in the general population. That is, in this situation the event of interest is only identifiable via a relative survival framework. When mortality is the endpoint the events that are not of primary interest (in this application those events that account for the remaining cancer consequent deaths) act as competing causes of death as they preclude the event of interest from occurring. To account for the competing causes we borrow ideas from classical competing risks theory when simultaneously modelling the component parts that make up the total excess mortality. For the current application we can re-express equation 3 in terms of the component parts of interest, i.e.,

(6) $$H(t)=H_{\text{cvd}}^{*}(t)+H_{\text{other}}^{*}(t)+\Theta {\left(t\right)}_{\text{cvd}}+\Theta {\left(t\right)}_{\text{other}},$$

where *other* is used to denote deaths due to other causes than CVD. Written on this form, $H_{\text{cvd}}^{*}(t)$ and $H_{\text{other}}^{*}(t)$ represent the expected mortality from CVD and other causes respectively and are assumed known from national mortality statistics. *Θ*(*t*)_*cvd*_ is our main quantity of interest and represents the excess CVD mortality rate among the patients whereas *Θ*(*t*)_*other*_represents the remaining excess mortality rate attributable to the cancer. The main contribution to *Θ*(*t*)_*other*_ comes from deaths from the underlying disease (breast cancer in this case), but side effects other than CVD, such as second malignancies contribute to the remaining excess hazard too.

Let j ∈{*cvdother*}. A competing risk analogue of the flexible parametric survival model for relative survival can now be written as

(7) $$\text{ln}(\Theta_{j}(t;x))=s(\text{ln}(t);\gamma_{0,j},k_{0,j})+x^{T}\beta_{j}$$

where $s(\text{ln}(t);\gamma_{0,j},k_{0,j})$ provides an estimate of the log cumulative baseline excess mortality for cause *j* and the vector ***β***_**j**_represents the covariate effects on cause *j*. We can fit separate models for each cause of death according to (7), and thereby allow the component-specific baseline cumulative excess mortality functions to take completely different shapes for the two outcomes. The analysis is standard if the research question is restricted to either *Θ*(*t*)_*cvd*_or *Θ*(*t*)_*other*_ or if both component parts are of equal interest but there are no joint parameters shared between the outcomes. The main requirement for fitting models of the type described in (7) is that the expected mortality files can be stratified by the causes of death in question. An equivalent approach to modelling multiple outcomes in one single step is however often required, in particular if we are willing to assume that some covariate effects are shared between the outcomes under study. However, modelling multiple outcomes simultaneously requires additional data preparation. In particular, the number of observations per individual in the data set need to match that of the number of causes of death under investigation (in our example, each woman will be represented by two rows in the new data set). In addition, a covariate representing cause of death is needed (in our example CVD or other) as well as a binary covariate indicating which of the different outcomes the individual eventually died from (if any). Data sets on this form are described in detail in [7] and are sometimes referred to a long format data sets. Having a data set up in long format allows the inclusion of a covariate representing cause of death as a time-dependent effect in the model and thereby the possibility to account for separate shapes for the underlying component-specific baseline excess mortality functions. To illustrate this in the case where only two competing causes of excess deaths are considered, we can express a joint model for the two outcomes as

(8) $$\begin{array}{cc} \text{ln}(\Theta_{j}(t;x)) & =s(\text{ln}(t);\gamma_{0},k_{0})+x^{T}\beta +c_{j}(\beta_{\text{cvd}}\\ \;+s(\text{ln}(t);\gamma_{\text{cvd}},k_{\text{cvd}})+x^{T}\beta_{\text{cvd}}) \end{array}$$

where

(9) $$c_{j}=\left\{\begin{array}{ll} 0, & \text{if}\;j=\text{other};\\ 1, & \text{if}\;j=\mathrm{CVD} \end{array}\right)$$

and where $s(\text{ln}(t);\gamma_{0},k_{0})$ now represents the log cumulative baseline excess mortality function for causes other than excess CVD, *β*_*cvd*_ the parameter that represents the shift in the baseline excess function if interest is in the excess CVD mortality, and $s(\ln(t);\gamma_{\text{cvd}},k_{\text{cvd}})$ the time-dependent effect that allows the baseline excess mortality function for the excess CVD mortality to vary freely. In this example, ^**x***T*^***β***denotes the effect of the covariates that are assumed common for the two causes whereas $x^{T}\beta_{\text{cvd}}$ represents interaction effects (i.e., the additional covariate effects for modelling the excess CVD mortality). Furthermore, additional complexity such as time-dependent covariate effects can easily be accommodated by including additional interaction terms with a spline term representing the underlying time scale (see equation 5 for details).

The individual contribution to the log likelihood for a flexible parametric model on the log cumulative hazard scale is described in detail in [20]. Stata’s **stpm2** module [20] was used to applying the proposed model to women with breast cancer in Sweden. The **stpm2** module is a readily available user-written program which uses Stata’s optimizer, **ml** (which in turn uses the Newton-Raphson algorithm) to maximize the likelihood function.

### Estimating crude treatment-related mortality

The crude probability of dying from cancer, *Cr*_*can*_(*t*), i.e., the probability of dying from cancer estimated in the presence of competing causes of death, can be calculated using standard competing risk definitions [7]. However, because we are in a relative survival setting, we first need to substitute the estimates of the all-cause survival, *S*(*t*) and cancer-specific hazard, *h*_*c*_(*t*), with their relative survival counterparts. This involves replacing *S*(*t*) with ^*S*∗^(*t*)*R*(*t*) and *h*_*c*_(*t*) with *λ*(*t*), respectively [14]. The crude probability of death from the cancer can hence be expressed as

(10) $$Cr_{\text{can}}(t)=\overset{t}{\underset{0}{\int }}S^{*}(u)R(u)\lambda (u)\mathrm{du.}$$

Similarly, the crude probability of death due to causes other than cancer, *Cr*_*non*−*can*_, is given by

(11) $$Cr_{\mathrm{non}-\text{can}}(t)=\overset{t}{\underset{0}{\int }}S^{*}(u)R(u)h^{*}(u)\mathrm{du.}$$

After having partitioned the excess mortality rate, *λ*(*u*), associated with cancer into component-specific excess hazard rates via (8), we can also partition *Cr*_*can*_(*t*) by extending (9). We are hence able to estimate the crude probability of dying from excess CVD (i.e. the treatment-related CVD), *C*_*rcan*,*cvd*_, as well as the remaining excess mortality attributable to the cancer, *Cr*_*can*,*other*_. Formally, this is achieved using

(12) $$Cr_{\text{can},j}(t)=\overset{t}{\underset{0}{\int }}S^{*}(u)R(u)\lambda_{j}(u)\text{du},\;j\in \{\text{cvd},\text{other}\}$$

where $R(u)=\underset{j}{\prod }R_{j}(u)$ and $\lambda_{j}(u)=\frac{d\Theta_{j}(t)}{\mathrm{dt}}$. The *Cr*_*can*,*j*_(*t*) can be viewed as marginal probabilities, i.e.,

(13) $$Cr_{\text{can}}(t)=\underset{j}{\sum }Cr_{\text{can},j}(t).$$

The integrands in equations 910 and 11 are non-linear functions of the model parameters and the integrals are obtained numerically by splitting the time scale into a large number, *n*, of small intervals and summing the values of the integrand for the *n* time intervals. 95% confidence intervals are retrieved using the delta method. For a detailed description of the method used for the numerical integration and for calculation of confidence intervals see Lambert *et al*[14].

### Application to breast cancer in Sweden - Description of the data

We obtained a data set encompassing all female breast cancer registrations in Sweden between 1 January 1973 and 31 December 1992 from the National Swedish Cancer Register [21]. All women had a potential follow-up of at least 15 years. Among the 70,655 women there were 8,939 deaths where the underlying cause of death was classified as CVD and 31,422 deaths where the underlying cause of death was classified as other than CVD. Cause-specific background mortality rates were created by combining publically available national mortality statistics with population-based information about the underlying cause of death reported to the Swedish Cause of Death Register 1973-2007. A detailed description of this procedure is provided in the appendix.

## Results and discussion

### Proportional excess hazards model

Time since diagnosis, estimated using survival times in days, was used as the underlying time scale. We fitted a proportional excess hazard model where age at diagnosis and calendar year of diagnosis were included as categorical variables. Interaction terms between age at diagnosis and cause of death and between calendar period and cause of death were included to allow the effect to differ for the two outcomes respectively. The log cumulative baseline excess mortality functions for both outcomes were modeled using restricted cubic splines with 5 df. The knots were places at the 0th, 20th, 40th, 60th, 80th and 100th centiles of the uncensored log event times. For comparison we also fitted a proportional excess mortality model for the total excess mortality (i.e., without partitioning the excess mortality into component parts). All reported p-values refer to results from likelihood ratio tests.

The estimated excess mortality rate ratios (EMRR) from the two models are shown in table 1. The EMRRs for the total excess mortality and the remaining (non-CVD) excess mortality are very similar. This is expected because the excess CVD deaths only constitute a relatively small portion of the total excess mortality. The effect of age at diagnosis on excess CVD mortality is more pronounced than for the excess remaining mortality (p for interaction < 0.001). The predicted excess CVD mortality rate for women aged 70-79 was 5.32 (95 % CI: 3.51-8.06) times higher than that of women aged 50-59 at diagnosis whereas the corresponding EMRR for the remaining excess mortality was 1.09 (1.04-1.14). There was no evidence against the hypothesis of a common effect of calendar period on the excess mortality for the two outcomes (p = 0.292).

**Table 1:** Parameter estimates from flexible parametric models

**Covariate**	**EMRR (CVD)**	**EMRR (remaining)**	**EMRR (total)**
< 50	0.40 (0.24-0.69)	0.96 (0.92-1.00)	0.95 (0.91-0.99)
50-59	1.00 (reference)	1.00 (reference)	1.00 (reference)
60-69	1.84 (1.16-2.91)	0.99 (0.95-1.03)	1.00 (0.96-1.04)
70-79	5.32 (3.51-8.06)	1.09 (1.04-1.14)	1.11 (1.06-1.16)
1973-1977	1.00 (reference)	1.00 (reference)	1.00 (reference)
1978-1982	1.13 (0.68-1.87)	0.82 (0.79-0.85)	0.82 (0.79-0.86)
1983-1987	1.23 (0.76-2.01)	0.76 (0.73-0.79)	0.78 (0.75-0.81)
1988-1993	0.76 (0.45-1.31)	0.58 (0.56-0.60)	0.59 (0.56-0.61)

Component-specific and total excess mortality rate ratios (EMRR) with 95 percent confidence intervals (CI) estimated from models assuming proportional excess hazards.

**Figure 1:**
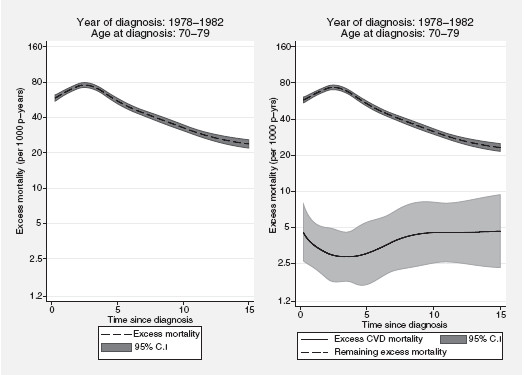
**Partitioning excess mortality.** Predicted total and component-specific excess mortality rates (per 1,000 person-years) estimated from a proportional excess hazards model among women diagnosed with breast cancer in Sweden at ages 70-79 years and between the years 1978-1982.

The left part of figure 1 shows the overall excess mortality rate per 1,000 person-years for patients diagnosed at ages 70-79 years between the years 1978-1982 as a function of years since diagnosis whereas the right part of the graph shows the mortality rates for excess CVD deaths and the remaining excess deaths separately. In cancer patient survival the overall cancer mortality rate is typically highest within the first few years after diagnosis. This is also observed in figure 1 although the pattern of the excess CVD mortality rate is somewhat different. In general, we expect to observe an increasing excess CVD mortality rate with increasing time since diagnosis. However, elderly women are more likely to be cardiologically fragile at the time of diagnosis which could potentially explain the high excess CVD mortality immediately following diagnosis. Because the main objective of this application is to study late adverse health effects of cardio-toxic therapies such as anthracyclines and radiotherapy we have chosen not to show any results for the first three months following the diagnosis of breast cancer.

### Non-proportional excess hazards models

**Figure 2:**
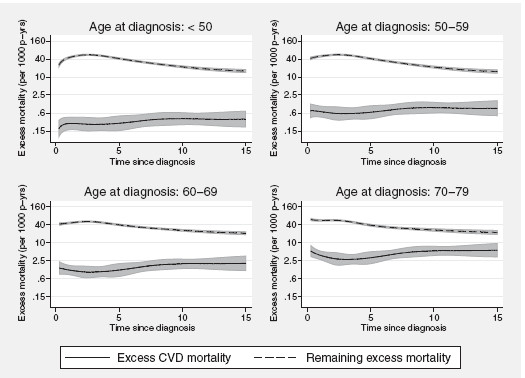
**Component-specific excess mortality rates.** Predicted component-specific excess mortality rates (per 1,000 person-years) among women diagnosed with breast cancer in Sweden between 1978-1982, estimated from a model accounting for time-dependent effects.

**Figure 3:**
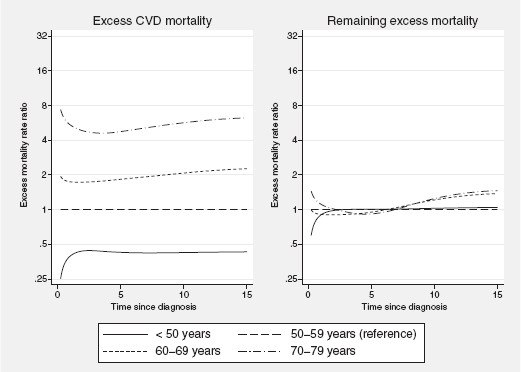
**Component-specific excess mortality rate ratios.** Predicted component-specific excess mortality rate ratios, (EMRR), among women diagnosed with breast cancer in Sweden between 1978-1982, estimated from a model accounting for time-dependent effects.

In cancer patient survival analyses it has previously been shown that the effects of age at diagnosis and calendar period are often non-proportional. The model was extended to evaluate the need for time-dependent effects for the two covariates by including additional interaction terms with the spline variables representing the time scale. Each time-dependent effect was modelled using 3 df (with knots placed at the 0th, 33rd, 67th and 100th centiles of the uncensored log survival times) whereas the baseline effect was still modelled using 5 df. In this model, the effect of calendar period was assumed common for the two outcomes. The resulting excess mortality rates per 1,000 person-years, for women diagnosed 1978-1982 are shown in figure 2. The excess CVD mortality tends to increase as a function of time since diagnosis whereas the opposite is seen for the remaining excess mortality. However, for the eldest age group, the excess CVD mortality seems to follow a U-shape the first 7 years after diagnosis which is probably reflecting that a selection of these patients are presenting with damaged vessels already at the time of diagnosis and are therefore at a higher risk for cardiac mortality even shortly after diagnosis. The excess CVD mortality rate increases with increasing age at diagnosis but is lower than the remaining excess mortality throughout follow-up. Figure 3 shows the predicted EMRRs for the effect of age at diagnosis on the component parts as a function of time since diagnosis. Apart from a tendency towards a departure immediately after diagnosis the effect of age at diagnosis on excess CVD mortality remains relatively constant throughout the 15 years of observation time (p for interaction = 0.346). A more notable departure from the proportional hazards assumption is observed for the remaining excess mortality, primarily among the two eldest age groups (p < 0.001).

**Figure 4:**
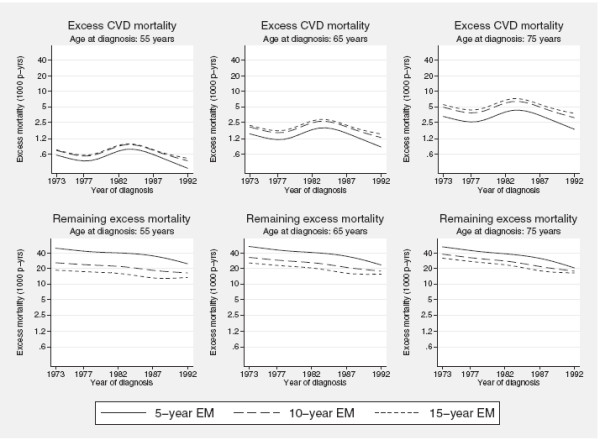
**Temporal trends in excess mortality rates.** Predicted 5-, 10- and 15-year component-specific excess mortality rates for women aged 55, 65 and 75 years respectively at diagnosis.

With the aim of studying temporal trends in excess mortality partitioned into component parts, we fitted a second non-proportional excess mortality model where age at diagnosis and calendar period were included in the model as continuous covariates using restricted cubic splines, each modelled with 4 df (with knots placed at the 5th, 25th, 50th, 75th and 95th centiles of the distributions of the two variables respectively). Both covariates were included as time-dependent effects (implying spline-spline interaction terms using 4 × 3 = 12 df to model the time-dependence for each of the two covariates). Neither the effect of age at diagnosis nor the effect of calendar year of diagnosis on the excess mortality rate were assumed to be common when modelling the component-specific excess mortality rates. In addition, the effect of calendar period on the remaining excess mortality was found to depend on age at diagnosis suggesting a need for a three-way interaction term in the model (p < 0.001). To reduce the potential risk of over parametrization the latter interaction term was restricted to the linear components (i.e., the first spline variable) of the spline terms representing age at diagnosis and calendar year. The predicted 5-,10- and 15 year excess mortality for the two outcomes for women aged 55, 65 and 75 years at diagnosis are shown as a function of year of diagnosis in figure 4. The bottom two graphs suggests a reduction in both short and long term remaining excess mortality over calendar time whereas no such trend is evident for the excess CVD mortality (top graphs). Although we lack global evidence of a statistically significant trend of a decreasing excess CVD mortality with calendar time, the tendency towards a reduced rate from the mid 1980’s can possibly be explained by an increasing use of partial mastectomies, resulting also in lower radiation doses to the heart [22].

### Crude probability of cancer death

**Figure 5:**
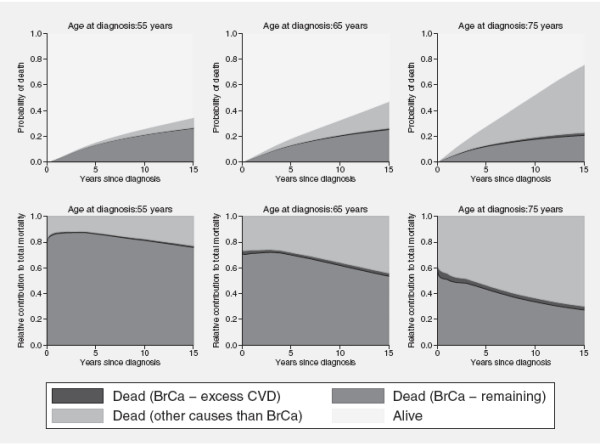
**Crude probabilities of death.** Predicted crude probabilities of death and the relative contribution of the component parts to total mortality among women diagnosed with breast cancer in Sweden in 1992 at ages 55, 65 and 75 respectively.

The crude probabilities of death due to cancer were partitioned by applying equation 11 to each component part after having partitioned excess mortality into component parts. The predicted relative survival estimates and excess mortality rates from the second non-proportional hazards model, described last in the previous section, were used in the numerical integration. The top three graphs in figure 5 show how the crude probabilities of death due to treatment-related CVD, breast cancer death (excluding the CVD deaths) and other causes respectively vary as a function of time since diagnosis for patients diagnosed in 1992 at ages 55, 65 and 75. The graphs clearly show how the risk of dying from any breast cancer related cause decreases as the patients are diagnosed at an older age whereas the risk of dying from causes other than the cancer increase with increasing age at diagnosis. For the two mutually exclusive sources of excess cancer mortality we see that the probability of death from excess CVD increased with age at diagnosis as opposed to the probability of death from the remaining cancer causes. This is due to treatment-related side effects being less common in younger ages when the patients are typically of better cardiac health.

The three bottom graphs of Figure 5 show the relative contribution of each component part, as well as death due to other causes, to the total mortality. Hence, by conditioning on that a woman, diagnosed in 1992 and aged 55, 65 or 75 years respectively at diagnosis, has died by time *t*, these graphs illustrate the proportion of all deaths estimated to be due to each possible cause of death. For all three age categories, the proportion of cancer deaths (excluding excess CVD deaths) decrease with elapsed time since diagnosis whereas the opposite is observed for deaths due to other causes than cancer. In contrast, the excess CVD deaths remain quite constant throughout the 15 years of follow-up.

**Figure 6:**
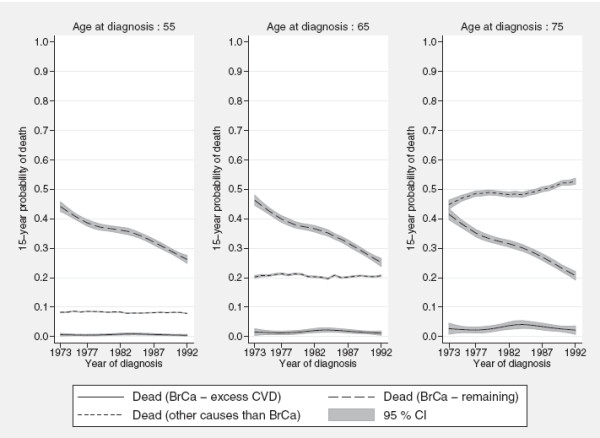
**Temporal trends in the crude probabilities of death.** 15-year crude probabilities of death among women diagnosed with breast cancer in Sweden at ages 55, 65 and 75 respectively.

Figure 6 summarizes temporal trends in the 15-year crude probabilities of death due to the three different causes of death for women of ages 55, 65 and 75 years at diagnosis estimated from the same model. For all three ages, dramatic improvements can be seen for the remaining excess cancer mortality whereas no improvement is observed for the excess CVD mortality. For example, the 15-year probability of death for women aged 65 years at diagnosis and who were diagnosed in 1973 is 0.016 (95% CI: 0.004,0.027) compared to 0.012 (95 % CI: 0.005, 0.019) for women diagnosed in 1992. Interestingly, the probability of death due to other causes does not seem to be affected by the substantial decrease in cancer mortality for women aged 65 years or younger at diagnosis. However, among the older women deaths due to other causes have increased somewhat during the period of of observation since the decreased cancer mortality has increased the ’opportunity’ to die from other causes.

### Assumptions and sensitivity analyses

There are two key assumptions for the proposed approach for partitioning the excess mortality into component parts. Firstly, although cause of death information is not used directly to identify patients who were reported to die from excess CVD (due to the inherent difficulty of determining whether the death would have occurred had the women not have been treated from breast cancer) it is used indirectly to determine the number of CVD deaths that occur in excess to what is expected in a population free of breast cancer. In order to study the impact of potential misclassification of CVD-events in the cause of death recordings obtained from Swedish official statistics we calculated the proportion of the observed CVD deaths that would have had to be coded erroneously in order to reduce the excess CVD mortality by 10, 15 and 20 percent respectively. The results, including also the observed and expected CVD counts in the data (stratified by age at diagnosis and year of diagnosis), are presented in table 2. The results show that, irrespective of covariate pattern, a misclassification of 6 to 7 % of the CVD deaths would decrease the excess CVD deaths by 10%. Nyström et al. [23] have previously examined the quality of the cause of death classification of 282,777 women (1,296 deaths) who participated in the Swedish randomized mammography trials between the years 1976 and 1982. The authors retrieved copies of medical records including autopsy protocols, death certificates, and histopathology reports and set up an end point committee to review the information relevant for assessment of the cause of death and found a high concordance concerning breast cancer as underlying cause of death with the information reported to the official statistics bureau in Sweden. The study supports the use of official health statistic in the evaluation of the Swedish screening trials. In this study cause of death was stratified on cardiovascular outcomes but the findings of Nyström et al. are nevertheless relevant even in this setting as they suggest that we are not likely to underestimate CVD deaths in this study as a consequence of breast cancer deaths being reported more frequently among the patients than they would in a disease free population. However, we recommend careful considerations of the quality of the national statistics prior to applying this method in other settings.

**Table 2:** Sensitivity of cause-of-death classification

	**At risk**	**Observed CVD deaths**	**Expected CVD deaths**	**Excess deaths (ED)**	**Proportion of misclassified CVD deaths required to reduce the ED:s by 10,15 and 20 %**		
					**10%**	**15%**	**20%**
Year of Diagnosis:							
1973-1977							
≤49 years	3339	35	11.9	23.1	6.6	10.0	13.1
50-59 years	3713	127	42.4	84.6	6.7	10.0	13.3
60-69 years	4522	600	205.3	394.7	6.6	9.9	13.2
70-79 years	4009	1313	621.8	691.2	5.3	7.9	10.5
Year of Diagnosis:							
1978-1982							
≤49 years	3447	28	10.5	17.5	6.4	9.3	12.5
50-59 years	3750	127	42.1	84.9	6.7	10.0	13.4
60-69 years	4933	607	219.4	387.6	6.4	9.6	12.8
70-79 years	4898	1592	733.7	859.3	5.4	8.1	10.8
Year of Diagnosis:							
1983-1987							
≤49 years	3894	29	9.5	19.5	6.9	10.0	13.4
50-59 years	3463	122	33.5	88.5	7.3	10.9	14.5
60-69 years	5055	571	203.1	367.9	6.4	9.7	12.9
70-79 years	5155	1554	696.3	857.7	5.5	8.3	11.0
Year of Diagnosis:							
1988-1992							
≤49 years	4640	44	12.2	31.8	7.3	10.9	14.5
50-59 years	4308	106	40.5	65.5	6.2	9.2	12.4
60-69 years	6200	598	240.6	357.4	6.0	9.0	12.0
70-79 years	5329	1485	630.9	854.1	5.8	8.6	11.5

The impact of misclassification of CVD deaths as non-CVD deaths among the cancer patients on excess mortality.

Secondly, relative survival analyses require the assumption that survival from the disease under study is independent of survival from other causes. The patients are also assumed to be exchangeable to the background population had they not been diagnosed with cancer. In the current study where we in fact study two outcomes in parallel the independence assumption is applied twice. Firstly, in order to accurately estimate excess CVD mortality among the patients we assume that the CVD risk among the patients (in the absence if cancer) is the same as the risk in the general population conditional on age, calendar year and sex. An equivalent assumption is also made for the remaining excess mortality. It is known, due to a differential distribution of risk factors, that the risk for breast cancer is greater among women from higher social classes, indicating that the distribution social class differs between patients and the general population. This suggests that the assumption of exchangeability may be violated as the background mortality rates are not stratified on social class. If this is the case the comparison population used is likely to have worse survival than what we would expect under independence. This could potentially bias the excess mortality rates for the component parts downwards suggesting that the observed estimates may, to some degree be, underestimated. It has, however, been demonstrated previously that such a bias is negligible in situations when the primary objective of the study is not to compare patient survival by social class [24].

**Figure 7:**
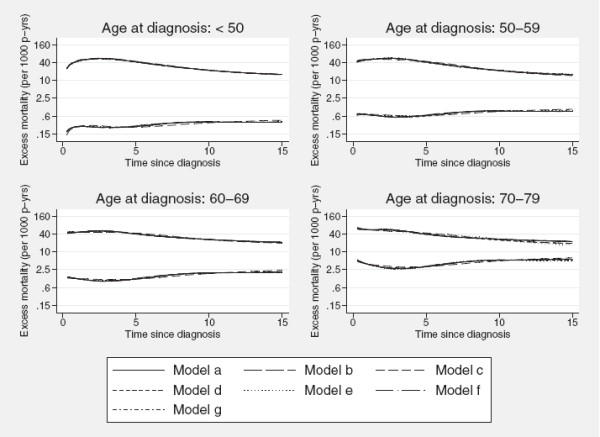
**Sensitivity to knot configuration.** Comparison of the predicted component-specific excess mortality rates (per 1,000 person-years) from models with varying number and location of the knots for the restricted cubic splines that represent the baseline excess mortality functions and time-dependent effects of age at diagnosis and calendar period.

Lastly, flexible parametric models use restricted cubic splines for modelling the log cumulative baseline excess mortality rate. Lambert et al. [14,20] have previously shown that the excess mortality rates are robust to the choice of number and placement of the knots used to define the spline terms. As a sensitivity analysis we fitted 6 models, all similar to the model described in the first part of section Crude probability of cancer death, to study the impact of the configuration of the knots used for modelling the component-specific baseline excess mortality rates as well as the restricted cubic splines involved in estimating the time-dependent effects of age and calendar year on the predicted excess mortality rates. Table 3 shows the distribution of knots used for each model (including model a) which generated figure 2) as well as the associated AIC (Akaike information criterion) and BIC (Bayesian information criterion). Varying degrees of freedom were used for the baseline log cumulative excess hazards, *df*_*b*_ and the time-dependent effects of the covariates, *df*_*t*_(covariate × time interaction). Figure 7 shows that the estimated excess mortality for the different models are very insensitive to the placement and number of knots used to model the spline terms. We do recognize from table 3 that the model a) is formally not the best-fitting model, based on the reported AIC or the BIC, but given the descriptive purposes of this application and the fact that the models provide extremely similar estimates of the excess mortality rates it was nevertheless used for demonstration of the method.

**Table 3:** Sensitivity to knot configuration

				**Model**	**Baseline, *df***_***b***_	**Age, *df***_***a***_	**Year, *df***_***y***_	**Number of parameters**	**AIC**	**BIC**
				a	5	3	3	38	304202	304537
				b	4	3	3	36	304219	304537
				c	3	3	3	34	304291	304592
				d	5	2	3	35	304207	304517
				e	5	4	3	41	304165	304527
				f	5	3	2	35	304197	304507
				g	5	3	4	41	304204	304565

Degrees of freedom for the baseline log cumulative excess mortality, *df*_*b*_, the time-dependent effect of age at diagnosis, *df*_*a*_and year at diagnosis, *df*_*y*_.

## Conclusions

We have shown how excess mortality due to cancer can be partitioned into component parts by fitting a flexible parametric survival model stratified on cause of death. The model is useful for simultaneously studying disease patterns and temporal trends for defined causes of cancer-consequent deaths. We have illustrated this by studying trends in the excess CVD mortality and remaining excess mortality among patients who have been exposed to a potentially cardiotoxic treatment following a diagnosis of breast cancer. These excess mortality rates quantify the transition rates to the events of interest in the situation where the patients are allowed to experience either of the events under investigation. The main advantage of modelling the different endpoints simultaneously is that, in contrast to fitting separate models for each outcome, it allows us to assume that the effects of some covariates are common for all outcomes. Moreover, likelihood-ratio tests or Wald tests may be used to formally assess this assumption [7].

In addition, we have shown how the model estimates may be used post-estimation to calculate crude probabilities of death due to the component parts. The two methods help to answer different questions but the crude probabilities of death will generally be of more interest to clinicians and patients when making decisions about treatments as these estimates provide an estimate of the probability of dying from, for example, treatment-related CVD in the presence of other causes of death.

### Future directions

The proposed model offers a possibility to monitor temporal trends in treatment-related excess mortality. From a public health perspective, being able to study if changes in clinical practise towards reducing treatment-related mortality have had an impact on patient survival is clearly of importance. In this article we chose to apply the method to study excess CVD among women with breast cancer, but we believe the basic idea of the method is useful also in other applications. Possible extensions of the method described in this paper include additional partitioning of the excess mortality. For example, an increased risk of lung cancer following treatment with radiotherapy has been reported among women with breast cancer [25]. Similarly, treatment for Hodgkin lymphoma has also been reported to increase the risk for secondary malignancies [26]. Finer partitioning of excess cancer mortality would, however, require additional use of the information stated on the death certificates and further work should address under what situations finer stratification is feasible. Moreover, because life-tables stratified on cause of death are typically not readily available, additional work examining the possible need for applying different smoothing techniques on the expected mortality rates might be of particular importance for studying rare outcomes.

Another interesting possible extension of the model would be to combine the proposed model with statistical cure models. Cure models within the framework of flexible parametric models have recently been proposed as an alternative to parametric cure models [27]. A limitation of cure models is that they are not appropriate unless long-term excess mortality tends to zero (i.e., a cure proportion exists). If the long-term excess mortality is due to one specific cause (e.g., excess CVD) then we can potentially partition out that component and subsequently fit a cure model. For example, combining the two proposed methodologies would allow for estimation of the a theoretical cure proportion after having partitioned out the excess mortality due to excess CVD mortality among patients diagnosed with lung cancer.

## Appendix

## Methods for generating life-tables stratified on cause of death

### Data

We obtained individual level data containing information about year of death, age at death, sex, underlying cause of death and any contributing causes of death for all people (n = 2,650,158) who died in Sweden between 1961 and 2007 and whose cause of death had been recorded as any disease of the circulatory organs. Cause of death is recorded in the Swedish official statistics according to the International Classifications of Diseases (ICD). For the current study the following ICD-codes were used to identify diseases of the circulatory organs, 

· ICD7(1961-1968):400-468,

· ICD8(1969-1986):390-458,

· ICD9(1987-1996):390-459 and

· ICD10(1997-2007):I00-I99.

The life-tables used in the analysis were stratified based on the underlying cause of death only, leaving 2,051,269 (77%) recorded cardiovascular events in the raw data. Of these, individuals older than 99 at the time of death were excluded (n = 520).

Data on the total number of deaths in Sweden as well as population counts, stratified on age, sex and calendar time was obtained in period format i.e., by year of occurence) from the Human Mortality Database (HMD) [28]. The HMD is a collaborative project sponsored by the University of Berkeley and the Max Planck Institute for Demographic Research. The raw data consist of birth and death counts from vital statistics plus population counts from official population estimates. A detailed documentation of the data cleaning [29] of raw data files is published on the HMD web-page [28].

### Calculation of cause-specific death rates

Data on population size (*N*), the total number of deaths (*d*) in Sweden and the total number of CVD deaths (*d*_*CVD*_) for the years 1973-2007 were collapsed over sex, age and calendar year. Probabilities of death due to CVD and non-CVD causes were estimated by taking the ratio of the death counts and population at risk in matched intervals of age (*i*), sex (*j*) and time (*k*). The corresponding mortality rates were then calculated using

(14) $$\lambda_{\text{CVD},i,j,k}=-\ln(1-\frac{d_{\text{CVD},i,j,k}}{N_{i,j,k}-\frac{d_{\text{CVD},i,j,k}}{2}})$$

and

(15) $$\lambda_{\text{non}-\text{CVD},i,j,k}=-\ln(1-\frac{d-d_{\text{CVD},i,j,k}}{N_{i,j,k}-\frac{\left(d-d_{\text{CVD},i,j,k}\right)}{2}}).$$

The cause-specific event rates were subsequently merged to the cancer patient data set (reshaped into long format) with respect to sex, age and year at each patient’s event time.

## Competing interests

The authors declare that they have no competing interests.

## Authors’ contributions

SE, PWD, PCL and TMLA conceived the project. SE carried out the analysis and extended the software to enable use of the method. All authors participated in the interpretation of the results. SE drafted the paper, which was later revised by all authors through substantial contributions to the contents of the paper. All authors read and approved the final manuscript.

## Pre-publication history

The pre-publication history for this paper can be accessed here:

http://www.biomedcentral.com/1471-2288/12/86/prepub
